# *QuickStats:* Age-Adjusted Percentages* of Adults Aged ≥18 Years Without a Usual Place of Health Care,^†^ by Region^§^ — National Health Interview Survey, 2017^^¶^^

**DOI:** 10.15585/mmwr.mm6813a5

**Published:** 2019-04-05

**Figure:**
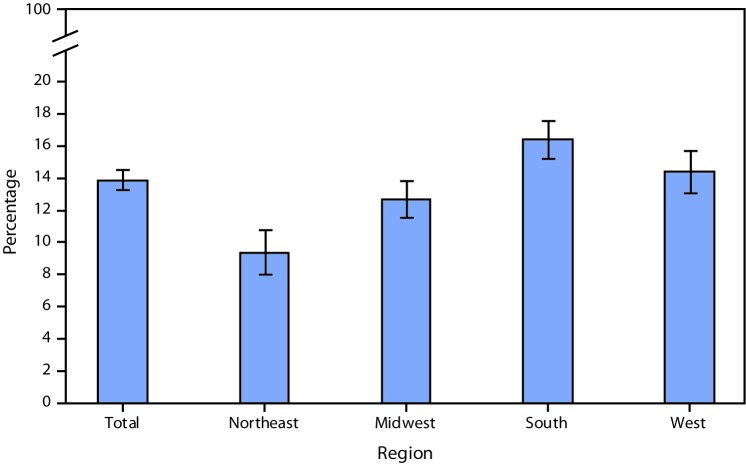
Among adults aged ≥18 years, 13.9% were without a usual place of health care in 2017. Adults in the South (16.4%) were more likely be without a usual place of health care compared with adults in the West (14.4%) and Midwest (12.7%). Adults in the Northeast (9.4%) were least likely to be without a usual place of health care.

**Source:** Tables of Summary Health Statistics, 2017. https://ftp.cdc.gov/pub/Health_Statistics/NCHS/NHIS/SHS/2017_SHS_Table_A-16.pdf.

